# Unclassified bronchopulmonary foregut malformation in a 2-year-old girl

**DOI:** 10.1186/s44215-023-00090-x

**Published:** 2023-08-04

**Authors:** Risa Kanai, Koji Fukumoto, Hiromu Miyake, Tatsuya Kawasaki, Katsumi Okuyama, Hideto Iwafuchi, Masashi Koyama

**Affiliations:** 1 Department of Pediatric Surgery, Shizuoka Children’s Hospital, Aoi-Ku, Shizuoka-Shi, Shizuoka, 860, Urushiyama 420-8660 Japan; 2 Department of Pediatric Critical Care, Shizuoka Children’s Hospital, Aoi-Ku, Shizuoka-Shi, Shizuoka, 860, Urushiyama 420-8660 Japan; 3 Department of Anesthesiology, Shizuoka Children’s Hospital, Aoi-Ku, Shizuoka-Shi, Shizuoka, 860, Urushiyama 420-8660 Japan; 4 Department of Pathology, Shizuoka Children’s Hospital, Aoi-Ku, Shizuoka-Shi, Shizuoka, 860, Urushiyama 420-8660 Japan; 5 Department of Radiology, Shizuoka Children’s Hospital, Aoi-Ku, Shizuoka-Shi, Shizuoka, 860, Urushiyama 420-8660 Japan

**Keywords:** Bronchopulmonary foregut malformation, Esophageal lung, Esophageal bronchus, Pulmonary sequestration, VACTERL association

## Abstract

**Background:**

Bronchopulmonary foregut malformation (BPFM) is a rare congenital anomaly characterized by a fistula between an isolated portion of respiratory tissue and the esophagus or stomach. Srikanth et al. reported that BPFMs can be categorized into four groups, and that an unclassified BPFM is extremely rare. Herein, we present an unclassified BPFM group III and IV subtype in a 2-year-old girl.

**Case presentation:**

At a gestational age of 36 weeks, a 1535-g female neonate was born as one of the dichorionic diamniotic twins. She had vertebral abnormality, anovestibular fistula, pulmonary artery sling, small right lung, mediastinal shift with dextrocardia, tracheal stenosis, and radial hemimelia. After birth, she was diagnosed with VACTERL association. She exhibited consolidation of the right upper lobe (RUL) in the neonatal period; however, she had no respiratory symptoms and was kept under observation in an outpatient visit. At 24 months, she was urgently admitted with acute pneumonia, and the upper gastrointestinal series revealed the right upper bronchus arising from the lower esophagus. Therefore, she underwent RUL resection. Intraoperatively, the right lung had no lobulations. RUL was ventilated by the esophageal bronchus (BPFM group III); however, the S6 lesion was ventilated by both the normal bronchial system and esophageal bronchus (close to BPFM group IV). The S6 lesion did not satisfy the definition of group IV as it did not have systemic blood supply. Hence, we decided to preserve the S6 lesion to save lung capacity as much as possible. The esophageal bronchus was transected using a 5-mm stapler. Due to lobulation failure, RUL was resected using an electric scalpel and 5-mm staplers along with the demarcation line by ventilation from the normal bronchus. The postoperative course was uneventful, and the patient could regain oral function without pneumonia or respiratory distress.

**Conclusions:**

For neonates with repeated consolidation and pneumonia, BPFM must be considered as one of the differential diagnoses. Regarding BPFM treatment, early recognition and imaging are necessary. To determine the resection area of the lung, it is crucial to consider the segment of ventilation from the normal bronchi.

## Background

Bronchopulmonary foregut malformation (BPFM) is a rare congenital malformation that involves both the digestive and respiratory systems [1, 2]. BPFMs can cause respiratory distress, cough while feeding, and recurrent pneumonia; however, a diagnostic delay remains common [1]. Srikanth et al. reported that BPFMs can be categorized into four groups [2]. Herein, we present an unclassified BPFM in a 2-year-old girl with VACTERL association.

## Case presentation

**Fig. 1:**
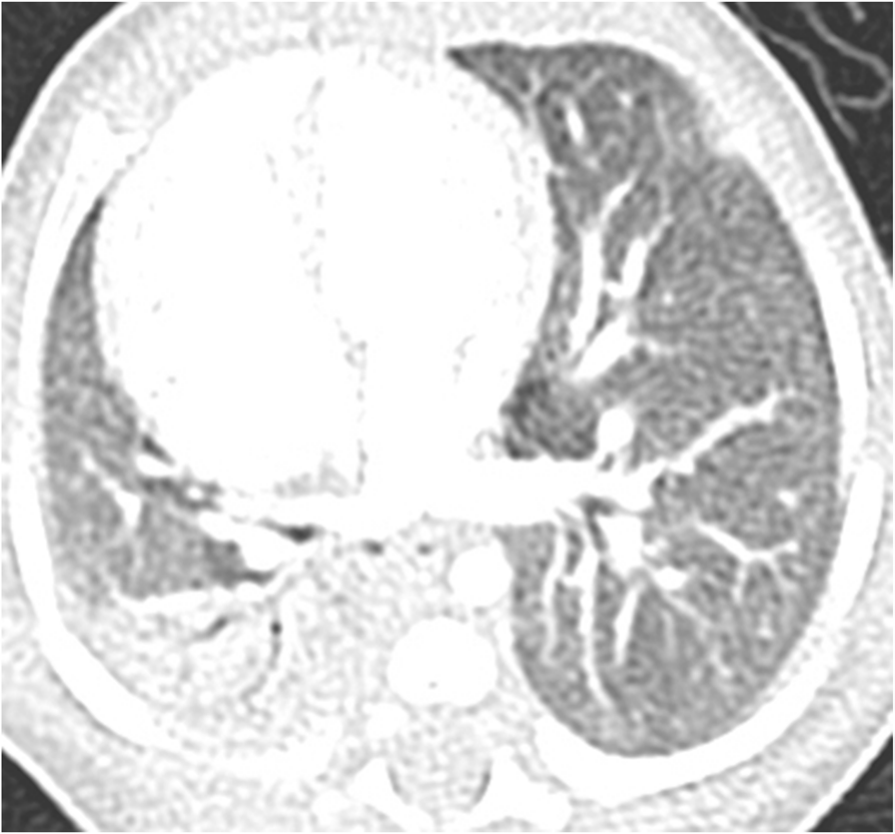
Computed tomography (CT) scan at 1 month of age. It revealed consolidation of the right upper lobe (RUL) and dextrocardia

**Fig. 2:**
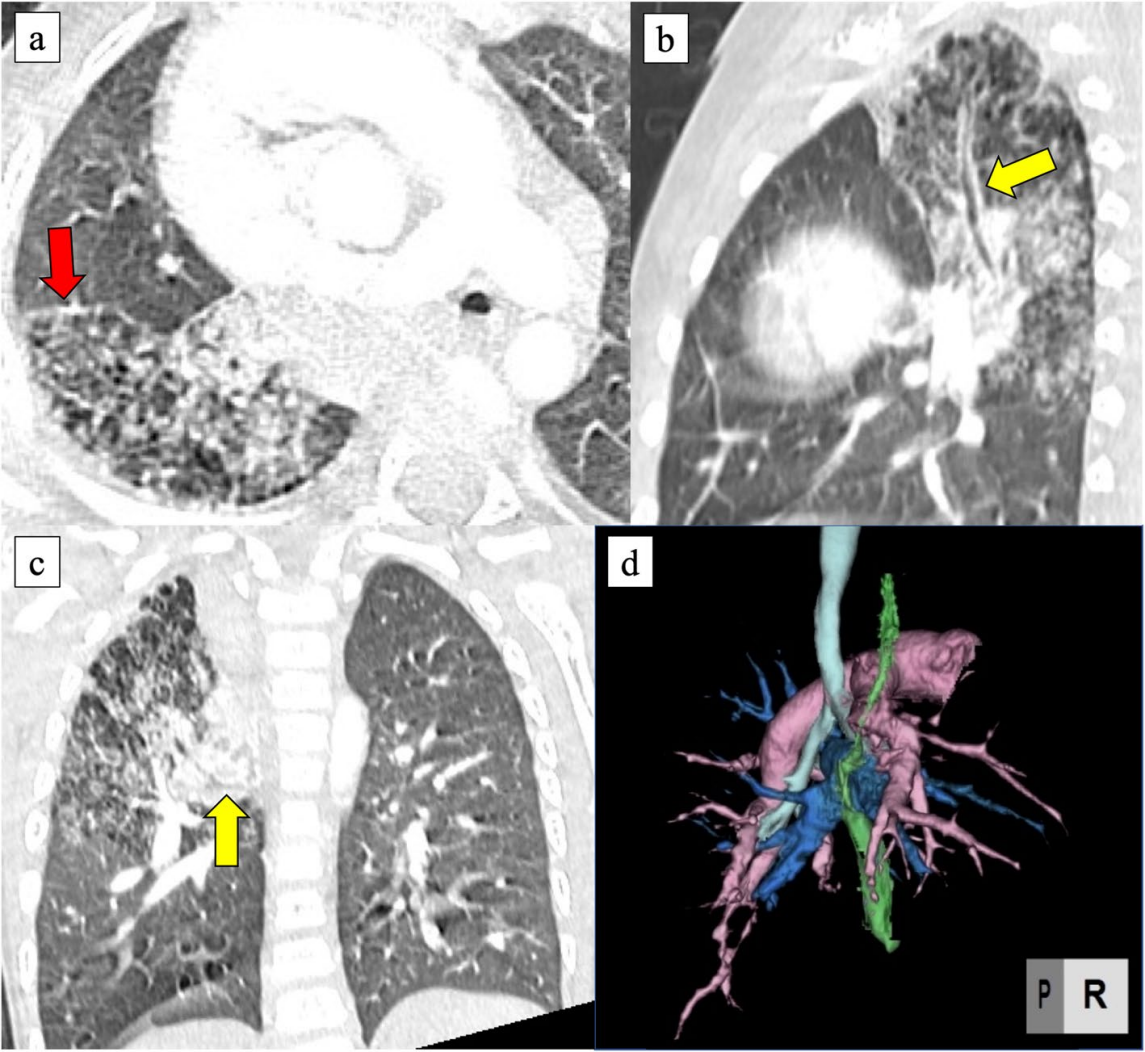
CT scan at 24 months of age. It revealed the aerated RUL with consolidation and esophageal bronchus (yellow arrow). The margin of the affected lung was seen (red arrow). **a** Axial, **b** coronal, and **c** sagittal sections. **d** 3-dimensional image. Her pulmonary artery and pulmonary vein in the right lung were of normal structure

**Fig. 3:**
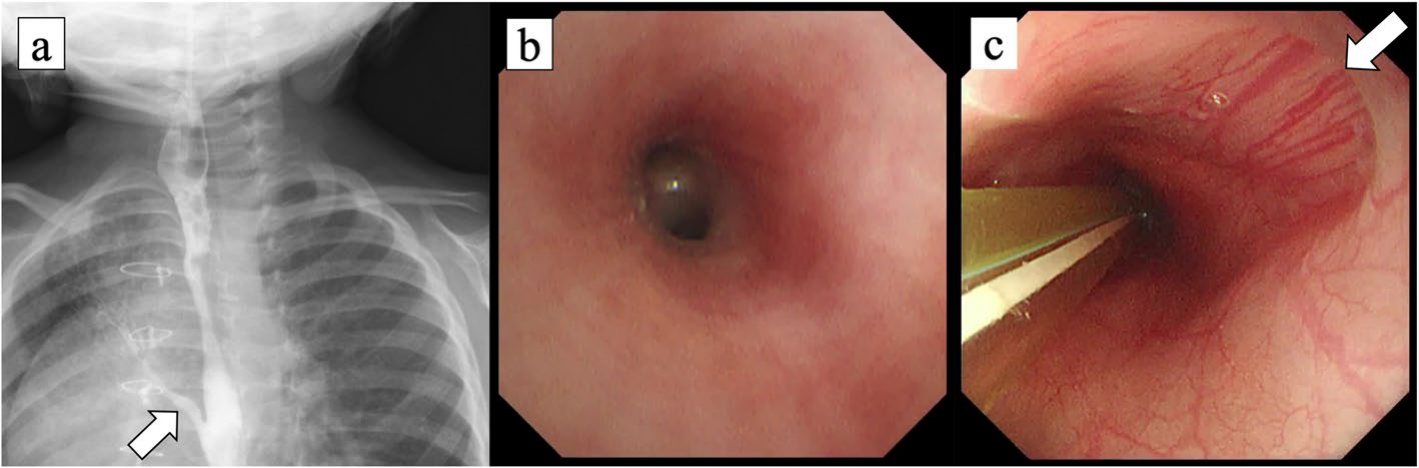
Esophageal bronchus to RUL. **a** The upper gastrointestinal series showed the right upper bronchus arising from the lower esophagus (arrow). **b** Bronchoscopy revealed that the right main bronchus was long and supplied two bronchi in the inner part. **c** Esophageal endoscopy revealed communication between the lower esophagus and the right upper bronchus (arrow)

At a gestational age of 36 weeks, a 1535-g female neonate was born as one of the dichorionic diamniotic twins. She had vertebral abnormality (V), anovestibular fistula (A), pulmonary artery sling, small right lung, mediastinal shift with dextrocardia (C), tracheal stenosis, and radial hemimelia (R). After birth, she was diagnosed with VACTERL association. In the neonatal period, chest X-ray imaging and computed tomography (CT) showed consolidation of the right upper lobe (RUL), and it was not improved by respiratory rehabilitation (Fig. 1). Esophageal bronchus was considered as one of the differential diagnoses, but was not strongly suspected because she had no respiratory symptoms while feeding. She was kept under observation in an outpatient visit. She underwent translocation of the right pulmonary artery for sling release at 6 months of age and underwent anoplasty at 11 months. Although intraoperative bronchoscopy could not identify the right upper bronchus, esophageal endoscopy also could not detect the esophageal bronchus. She did not develop pneumonia and recovered smoothly after these operations. However, at 18 months, she began to present occasional postprandial cough. At 24 months, she was urgently admitted with acute pneumonia. CT revealed the aerated RUL with consolidation (Fig. 2). The upper gastrointestinal series (UGI) showed the right upper bronchus arising from the lower esophagus (Fig. 3a). Bronchoscopy revealed that the right main bronchus was long and supplied two bronchi in the inner part (Fig. 3b), and esophageal endoscopy revealed this communication (Fig. 3c). In enhanced CT, the right lung received pulmonary but not systemic blood supply.

**Fig. 4:**
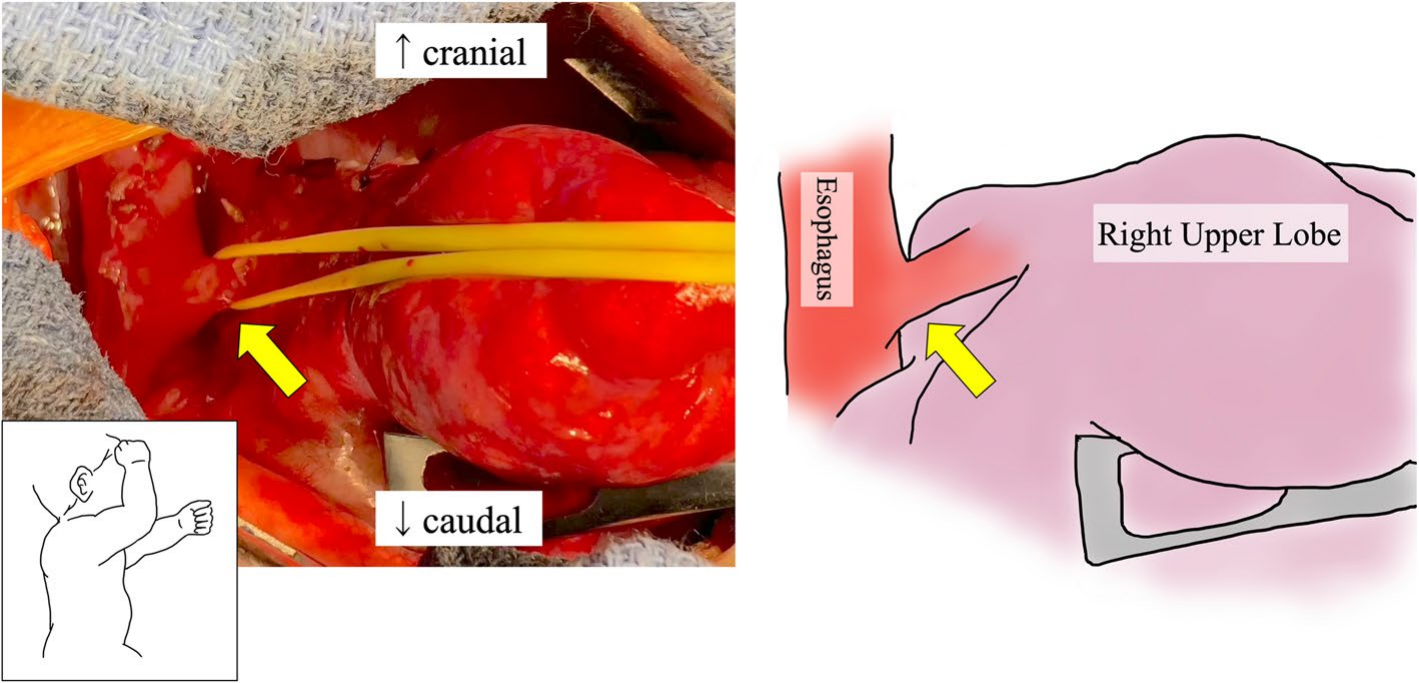
Intraoperative findings. The esophageal bronchus (yellow arrow) to RUL could be seen

**Fig. 5:**
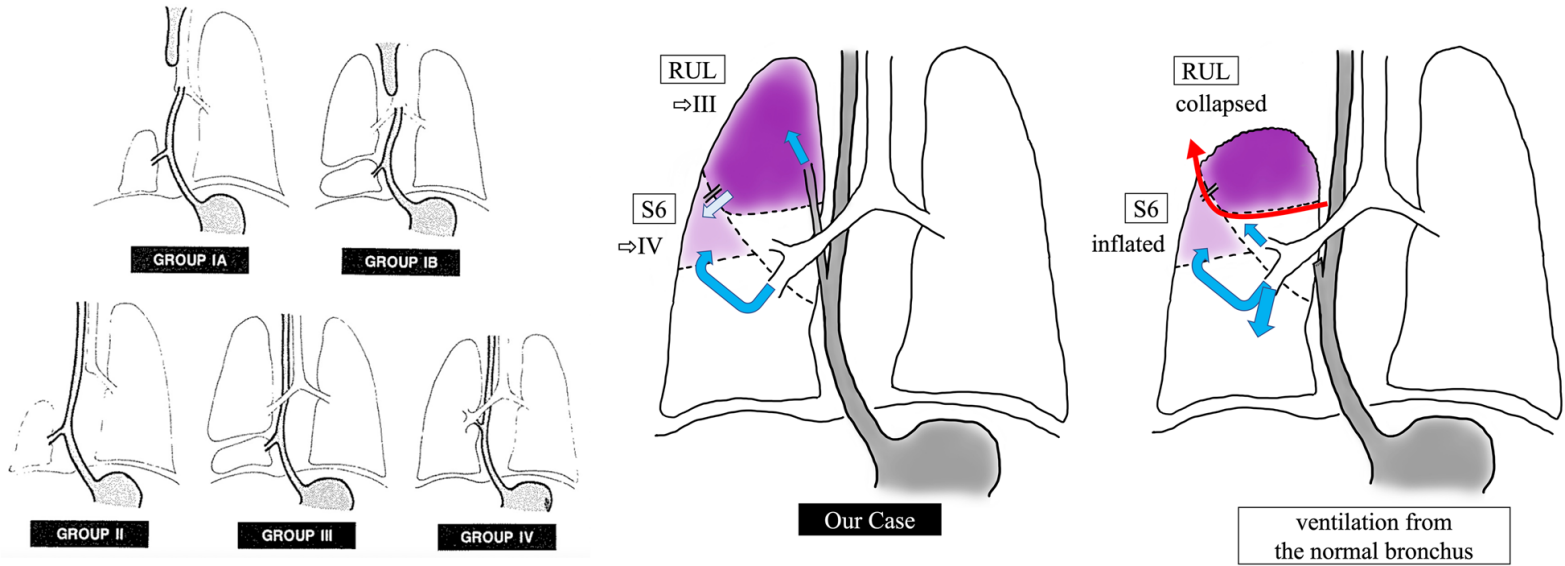
Schemas for BPFMs. Citation from *Srikanth *et al*.*^2^. BPFMs are categorized into four main groups. In our case, the right upper lobe was ventilated by the esophageal bronchus (BPFM group III); however, the S6 lesion was ventilated by both the normal bronchial system and esophageal bronchus (close to BPFM group IV). The ventilation is indicated by blue arrows. We transected the esophageal bronchus. Via ventilation from the normal bronchus, RUL collapsed; however, the S6 lesion inflated. Due to lobulation failure, we resected RUL using an electric scalpel and 5-mm staplers along with the demarcation line (red arrow). We hypothesized that the S6 lesion and the RUL have peripheral bronchial communication with each other (light blue arrow)

**Fig. 6:**
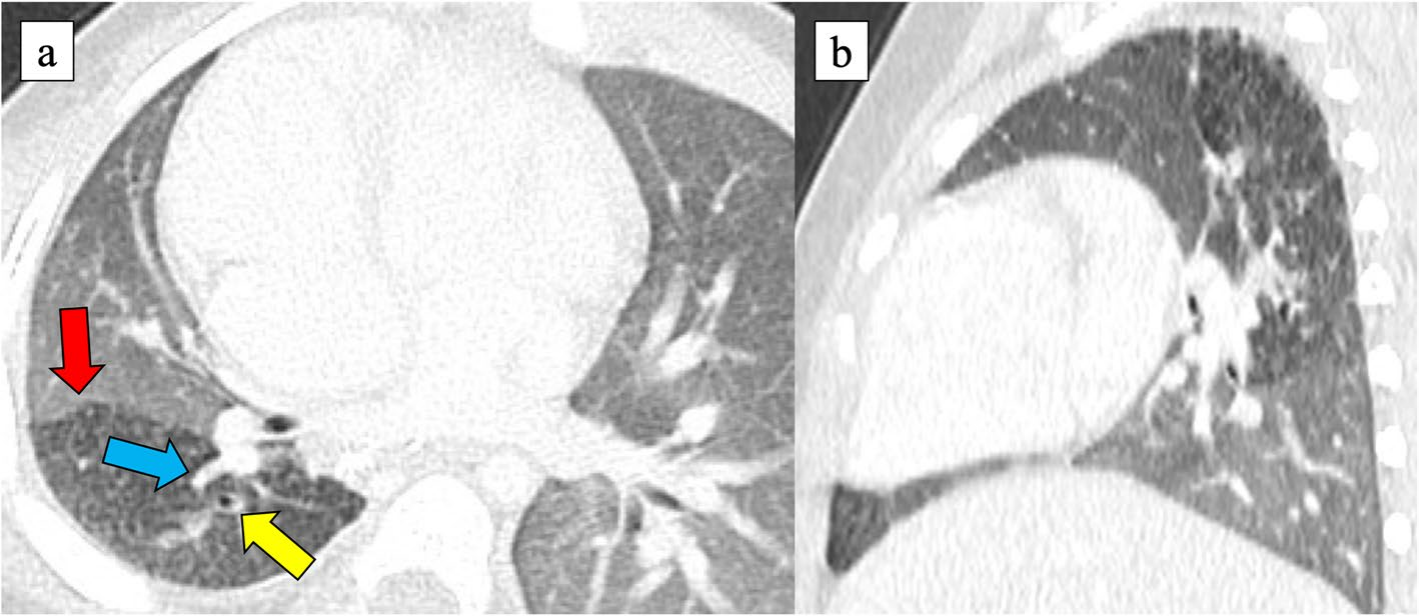
CT scan 3 months postoperatively. It revealed slight overinflation of the preserved affected lung (the S6 lesion) with pulmonary vessels (V6: blue arrow) and bronchus (B6: yellow arrow). The margin of the affected lung could be seen (red arrow). **a** Axial and **b** sagittal section

At thoracotomy, the right lung had no lobulations. The RUL was obviously firm and could be identified as an affected lesion; however, the firm lesions seemed much more extensive. The lobulation failure, a small working space due to dextrocardia, and postoperative adhesion around the right pulmonary artery rendered the operation complicated. As there was no space between the lobes, we started to approach the ventral pulmonary hilum. The pulmonary vessels of A1 + 3 and V1–3 were transected. Subsequently, we gradually cut the lung parenchyma between V3 and V4 along the main pulmonary artery trunk. The pulmonary artery of AscA2 was identified and transected. In the dorsal approach, we identified the esophagus and the esophageal bronchus raised from the lower esophagus (Fig. 4). The esophageal bronchus existed apart from the normal right bronchus. We transected it using a 5-mm stapler. We might have been better off flipping the esophageal mucosa internally to prevent recurrence. On the other hand, it was difficult because of poor surgical vision in a pediatric patient. The demarcation line of the affected lesions outside the RUL was ambiguous due to lobulation failure, and then, we clarified the line via ventilation from the normal bronchus. As a result, RUL collapsed; however, a part of the firm affected lung obviously inflated. We decided to preserve this inflated segment to save lung capacity as much as possible and resected RUL using an electric scalpel and 5-mm staplers along with the line (Fig. 5). Histological findings in the resected RUL showed a mixture of emphysematous changes and obstructive changes with inflammatory cell infiltration and necrosis. The alveolar architecture was indistinct and was occupied by cystically dilated air spaces. The postoperative course was uneventful, and the patient could regain oral function without pneumonia or respiratory distress. After 3 months, postoperative CT revealed that the preserved segment was slightly overinflated, but not necrotic. The margin of the affected lung persisted (Figs. 2a and 5a). The preserved segment included pulmonary vessels of A6 and V6 and bronchus of B6 (Fig. 6), and it was recognized as S6 lesion. After much discussion, the patient was finally diagnosed with unclassified BPFM group III (RUL) and group IV subtype (S6 lesion).

## Discussion and conclusions

BPFMs are rare congenital anomalies characterized by a fistula between an isolated portion of respiratory tissue and the esophagus or stomach. They are caused by embryological faults of the lung and esophagus and often related to esophageal atresia, tracheoesophageal fistula, pulmonary sequestration, cardiovascular anomalies, and VACTERL association [1]. BPFMs can involve any lobes, most frequently unilateral whole lobes or lower lobes. Upper lobes are relatively rare for BPFMs [2–4].

Most BPFM cases are diagnosed in the neonatal period; however, a diagnostic delay remains common [1]. Some cases were diagnosed in adulthood. For neonates with repeated consolidation and pneumonia, BPFM must be considered as one of the differential diagnoses, especially in children with malformation syndromes. When BPFM is suspected, immediate UGI is the first choice to delineate the abnormal bronchus connected to the esophagus [5]. UGI revealed a high diagnostic ability compared with bronchoscopy or esophageal endoscopy [1]. In our case, we should have performed UGI much earlier. If esophageal bronchus exists, surgery should be considered as soon as possible. Diagnostic delay can cause secondary destruction of the other healthy lobes [4]. In most cases, resection of abnormal pulmonary tissues is preferred, and reconstruction procedures are feasible in selected patients [1, 6, 7].

According to Srikanth et al., BPFMs can be categorized into four main groups: (Fig. 5) [2] group I is associated with esophageal atresia and tracheoesophageal fistula, group II has a unilateral whole lobe originating from the lower esophagus, group III has an isolated anatomic lung lobe or segment that communicates with the esophagus or stomach, and group IV has a portion of the lung ventilated by the normal bronchial system and esophageal bronchus with systemic blood supply [2]. In our patient, the upper lobe was ventilated only by the esophageal bronchus (BPFM group III); however, the S6 lesion was ventilated by both the normal bronchial system and esophageal bronchus (close to BPFM group IV). The S6 lesion did not satisfy the definition of group IV as it did not have systemic blood supply. The other hypothesis is that repeated infections of RUL destroyed the S6 lesion which was ventilated only by the normal bronchus. Considering the postoperative slight overinflation of the S6 lesion, we speculated that the S6 lesion and RUL have peripheral bronchial communication with each other (Fig. 5). After the surgery, some of the air escape routes to RUL were eliminated and overinflation occurred in the S6 lesion. Histological findings in the resected RUL showed a mixture of emphysematous changes and obstructive changes with inflammatory cell infiltration and necrosis. The alveolar architecture was indistinct and was occupied by cystically dilated air spaces. The parenchyma of the S6 lesion may have similar histological changes and have been more easily inflated than the other normal lobes. It remains unclear how the S6 lesion was preoperatively ventilated. It may have to be resected if it is affected by pneumonia in the future.

The patient was finally diagnosed with unclassified BPFM group III and IV subtype. Congenital lobulation failure or secondary destruction from the upper lobe may have been related to the unclassified BPFM situation of the patient. To the best of our knowledge, an unclassified BPFM is rarely reported [4, 8]. Trisno et al. reported an unclassified BPFM case in which repeated infections of the right lower lobe destroyed the other right lobes [4]. John et al. reported a case of group IV subtype that received pulmonary blood supply alone [8]. We could only find these two cases of unclassified BPFM, and we also consider our case as extremely rare.

For neonates with repeated consolidation and pneumonia, BPFM must be considered as one of the differential diagnoses, especially in children with VACTERL association. When BPFM is suspected, immediate UGI plays a significant role in its diagnosis. If esophageal bronchus exists, surgery should be considered immediately. In determining the resection area of the lung, it is crucial to consider the segment of ventilation from the normal bronchi, although an unclassified BPFM is extremely rare.

## Data Availability

The datasets supporting the conclusions of this article are included within the article.

## Abbreviations

BPFM: Bronchopulmonary foregut malformation
RUL: Right upper lobe
CT: Computed tomography
UGI: Upper gastrointestinal series
